# OPTYRE—Real Time Estimation of Rolling Resistance for Intelligent Tyres

**DOI:** 10.3390/s19235119

**Published:** 2019-11-22

**Authors:** Nicola Roveri, Gianluca Pepe, Federica Mezzani, Antonio Carcaterra, Antonio Culla, Silvia Milana

**Affiliations:** Department of Mechanical and Aerospace Engineering, Sapienza University of Rome, 00184 Rome, Italy; gianluca.pepe@uniroma1.it (G.P.); federica.mezzani@uniroma1.it (F.M.); antonio.culla@uniroma1.it (A.C.); silvia.milana@uniroma1.it (S.M.)

**Keywords:** rolling resistance, fibre Bragg grating, intelligent tyre

## Abstract

The study of the rolling tyre is a problem framed in the general context of nonlinear elasticity. The dynamics of the related phenomena is still an open topic, even though few examples and models of tyres can be found in the technical literature. The interest in the dissipation effects associated with the rolling motion is justified by their importance in fuel-saving and in the context of an eco-friendly design. However, a general lack of knowledge characterizes the phenomenon, since not even direct experience on the rolling tyre can reveal the insights of the correlated different dissipation effects, as the friction between the rubber and the road, the contact kinematics and dynamics, the tyre hysteretic behaviour and the grip. A new technology, based on fibre Bragg grating strain sensors and conceived within the OPTYRE project, is illustrated for the specific investigation of the tyre dissipation related phenomena. The remarkable power of this wireless optical system stands in the chance of directly accessing the behaviour of the inner tyre in terms of stresses when a real-condition-rolling is experimentally observed. The ad hoc developed tyre model has allowed the identification of the instant grip conditions, of the area of the contact patch and allows the estimation of the instant dissipated power, which is the focus of this paper.

## 1. Introduction

The automotive industry is one of the major actors in the stage of greenhouse emission and electricity production [1] and the enhancement of road vehicle efficiency becomes the answer to the environment-wise concern. In this regard, the rolling resistance plays a key role among many sources of dissipation, since it can cause up to 30% of the fuel consumption, according to the driving regimes [2].

Analogous to a drag force, the resistance occurring during rolling is the expended energy in a unit of travelled distance. Tyres are made of a viscoelastic material, i.e., by reinforced rubber, and undergo phenomena of hysteresis. During loading and unloading phases, the stiffness curves of the tyre do not exactly match, producing energy loss: the more a tyre deforms, the higher the amount of generated heat. In addition, hysteretic effects depend on the mechanical characteristics of the tyre; for instance, as a counterintuitive phenomenon, the dissipated energy decreases as the temperature increases. This aspect explains the reason why the design of an intelligent tyre [3,4,5,6] finds its origin and its foundations also in the comprehension of the energy loss mechanisms and suggests the importance of experimental campaigns aimed at measuring the rolling resistance. The optimal design of a tyre must balance several, often discordant, requirements [7], such as adequate handling abilities, with a good level of grip for manoeuvring vehicles during cornering, braking and acceleration, while still keeping low the level of dissipated energy.

The development of an embedded system of sensors [8,9] for monitoring key variables, such as pressure, strain, temperature, acceleration, wheel loading, friction and tread wear, requires advanced technologies in the field of sensors and data transmission systems. In addition, these last need a dedicated power supply system, itself a challenging task [10]. This demands the development and the introduction of advanced technologies for any stage of the setup chain. In the last decade, conceiving a tyre as a sensor has been the ultimate goal of part of the automotive technologies, mainly targeted to friction identification. Indirect methods are devoted to extracting, for instance, the wheel angular velocity or the vehicle speed [11,12] from the acquired parameters. The nonlinear relationships [13,14,15] relating tyre parameters demand the employment of either Kalman filter [16] or fuzzy logic controllers [17]. Indirect methods, even though their hardware apparatus can be easily installed, lack high accuracy, and a calibration procedure is needed at any tyre change and at any pressure adjustment [16].

There are several other methods, other than the indirect techniques, that take advantage of direct observations with higher accuracy. Among them, Micro electro-mechanical systems (MEMS) are the most commonly used as pressure sensors [18] and for tread deformation acquisition [19], standing the fact that sensitivity is strictly related to the particle size. Empirical models [20] are tools used to characterize the relationship between rolling resistance and vehicle speed, even though its online measurement has not been directly investigated yet.

Spatial and time accuracy are not the most remarkable features of the data acquired through the above-mentioned methods and rapidly varying dynamic parameters are hard to be sensed and transmitted [21]. Further improvements in digital electronics and signal analysis processing are required to achieve more reliable resolution. The absence of commercial sensors for the direct acquisition of the tyre–road grip conditions confirms these difficulties. There are only few examples potentially resulting in industrial products, based on advanced sensors and electronic systems for real-time estimation [8,9,22], as for instance the Cyber Tyre [9,23]. However, the actual application of this sensing system is limited to the tyre internal pressure and contact patch monitoring, for which much simpler systems can be installed (based for example on the deviation of the rolling speed of one considered wheel with respect to the average of the others, as, for example, using the flat tyre monitoring (FTM) system).

It becomes clear how sensing systems, provided with simple architecture, and accurate identification methods [24,25,26] represent an urgent goal. In this context and in the general frame of a wider project [7,8,21,22], the ambition of this work was to develop a system enabling the tyre grip evaluation and the real-time identification of the stress of a tyre during the rolling regime. The strain inside the tyre was monitored by an ad hoc optical system, integrated into the tyre itself; based on these data, the analytical model manipulated the strain and returned, in real-time, the estimated rolling resistance.

The OPTYRE grip sensor is part of the Sapienza University of Rome’s autonomous vehicle project, "Auto Sapiens", developed by the Vehicle Dynamics and Mechatronics Lab team. The OPTYRE provides in real-time the state of the grip of the tyre to control the vehicle during braking, acceleration and during cornering manoeuvres and allows excellent safe driving performance using suitable controllers to be achieved [27,28,29,30,31].

The paper is organized into four main sections. Section 2 describes the experimental setup. Section 3 introduces a tyre analytical model on which the algorithm designed to evaluate the rolling resistance is based. This model permits results of the experimental campaign, discussed in Section 4, to be obtained and the real-time rolling resistance identification procedure. Eventually, Section 5 portrays the conclusions and outlines future developments.

## 2. The Layout of the OPTYRE System

This section describes the measurement scheme and the experimental setup, starting from the fibre Bragg grating (FBG) sensors embedded inside the tyre. 

The FBG based sensors, composed of a single-mode optical filter, present a spatially recurring glass refraction index. Because of this grating, the fibre becomes an optical filter: when infrared light is sent into the fibre, only a specific narrow band is reflected back towards a spectrum analyser. The remaining light is transmitted undisturbed along the optical waveguide.

**Figure 1:**
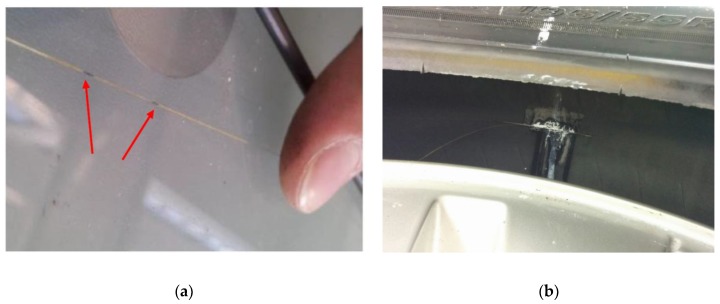

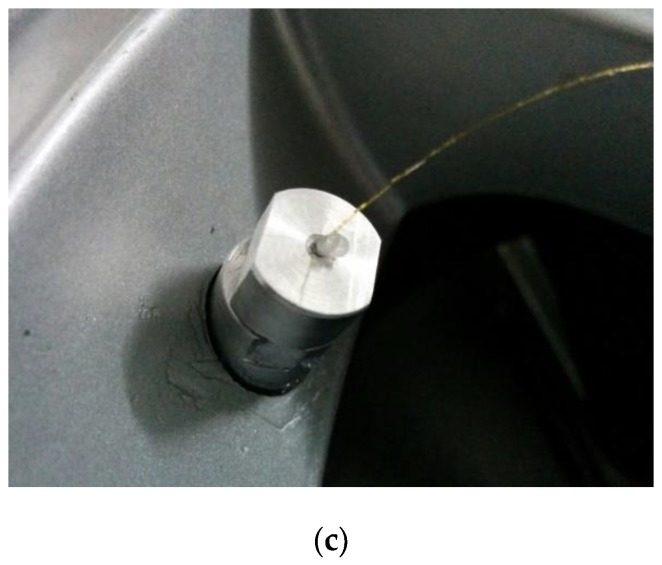
(**a**) The optical fibre with one fibre Bragg grating (FBG), the sensor lies within the black marks on the line; (**b**) the optical fibre pasted into the inner liner of the tyre; (**c**) the fibre exit connector from the rim.

This makes the FBG a distributed Bragg reflector [32], shown in Figure 1a. Indeed, the periodical variations of the refraction index in the optical fibre are arranged in a way that only specific wavelengths of light are reflected, transmitting all the others, which follow freely their path. This discloses a strict connection between the reflected Bragg wavelength λ*_B_*, the refractive index *n* of the fibre and the period of the refractive index modulation Λ: λ*_B_* = 2*n*Λ. The FBG turns into a strain sensor able to detect local axial deformation of an optical fibre. The previous expression clearly states how the reflected wavelength λ_B_ is sensitive to any modification of the grating sensors properties. Among them, the temperature variations change *n* as a consequence of the thermo-optic effects; Λ as well varies with the thermal modification if the fibre is unconstrained; in the same way, deformation and strain of the fibre cause alteration of Λ and *n*. This behaviour is completely described by Equation (1) in which the two terms on the right-hand side are related to the effects on λ*_B_* due to variations of the strain and the temperature, respectively.

Accurate monitoring of the variations of the wavelength of the FBG sensors allows the estimation of these perturbations. The simultaneous presence of strain and temperature excursion demands two FBG sensors for the acquisition: one must be in-built to the structure to acquire the strain, the other, dedicated to temperature recording, positioned close the first one, must be kept detached from the structure, to avoid the influence of the strain. 

FBG sensors present several advantages, reasons for the increasing interest they enjoy and the number of applications that see them involved, especially on structural health monitoring [33,34]. Data acquisition in large areas is enabled simply by embedding a large number of sensors on a single multiplexed fibre. Finally, FBG sensors do not suffer electromagnetic interference, have compact dimensions, do not need a dedicated power supply system: the light beam is the signal carrier and the power line at the same time.

In this particular circumstance, the idea was to place a number of optical fibres, equipped with several FBG sensors, along the inner circumference of the tyre. The case here investigated saw a single FBG sensor, Figure 1a, embedded on the inner surface of the tyre [8], Figure 1b, through the connection valve installed on the rim as depicted in Figure 1c.

**Figure 2:**
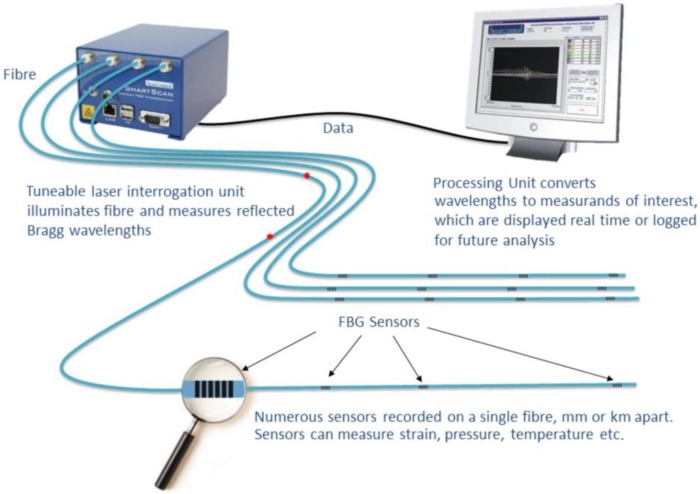
Measurement scheme of the FBG sensors.

Figure 2 depicts the measurement scheme described ahead:

- An optical led source produced the light beam, whose wavelength belonged to the far-infrared range; the path of the light beam was the optical fibre that, equipped with several FBG sensors, was attached to the circumference of the tyre.
- A spectrum analyser acquired the variations of the reflected light frequency bandwidth, caused by deformations of the tyre.
- The computer received and processed the signal sent by the spectrum analyser, which has the function to suitably sample the incoming analogue data.

Once the instant wavelength of each FBG sensor is detected by the spectrum analyser, the local strain *ε* is evaluated through:

(1) $$\varepsilon_{j}\left(t\right)=K_{\varepsilon }\frac{\lambda_{j}\left(t\right)-\lambda_{0j}}{\lambda_{0j}}-\alpha \Delta T$$

which relates the instantaneous wavelength $\lambda_{j}$ of the *j*-th FBG sensor to the strain. In Equation (1), $\lambda_{0j}$ is the reference wavelength,$\;K_{\varepsilon }=1.27$ is a gain factor, ΔT is the temperature variation, which is roughly zero in our tests, and α is the thermal expansion coefficient of the optical wire.

**Figure 3:**
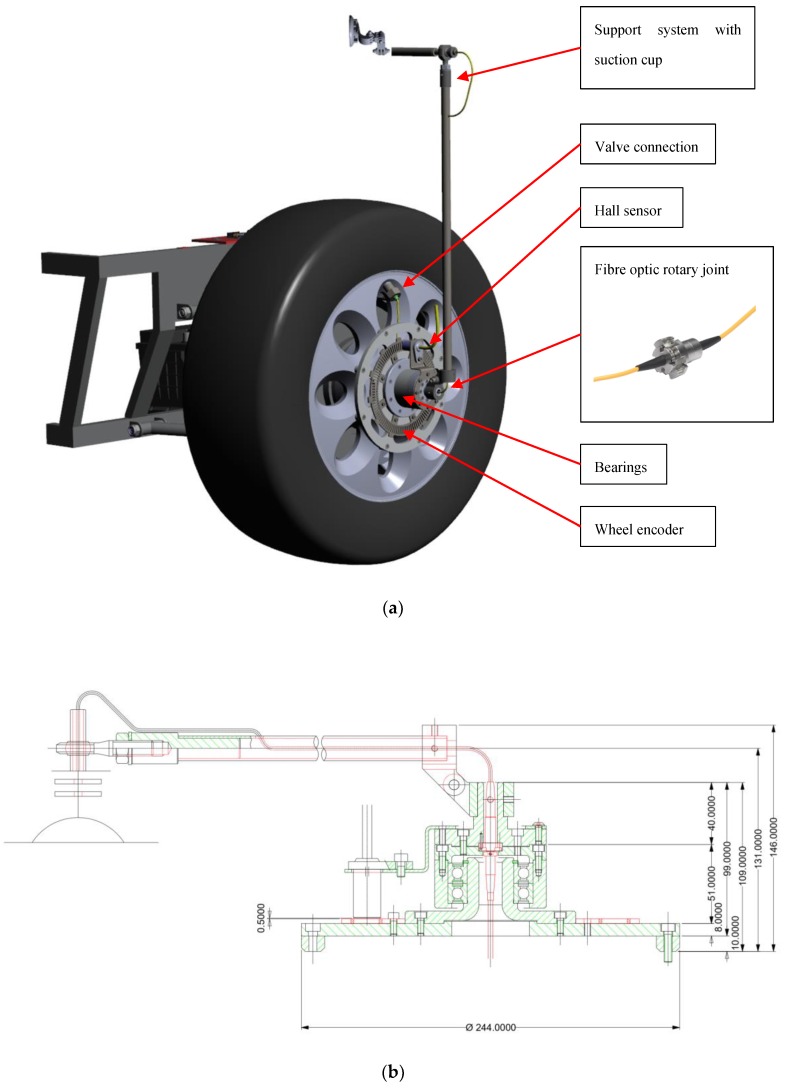
(**a**) The final assembly CAD of the wheel sensor device; (**b**) the side view CAD of the wheel sensor device with the FBG rotary coupler.

Since the FBG interrogator was integral to the car chassis, a fibre optic rotary coupler, inserted between the interrogator and the tyre as in Figure 3a,b, was necessary to acquire data during dynamic operations. The major complication stands in the data transmission, which must be ensured even during rotations. This required a device, the rotary joints in this particular case, able to communicate the optical signal across rotating surfaces. These rotary joints performed the same function of electric slip rings, guaranteeing the transmission of electric signals. The special application demanded an ad-hoc design of the coupler, presented in Figure 3a, which considers a static flange linked to the car chassis, and a rotating flange, coaxial with the tyre axes and connected with the rim. Eventually, a coupler case had the double function of connection to the wheel and protection for rotary joints from debris.

**Figure 4:**
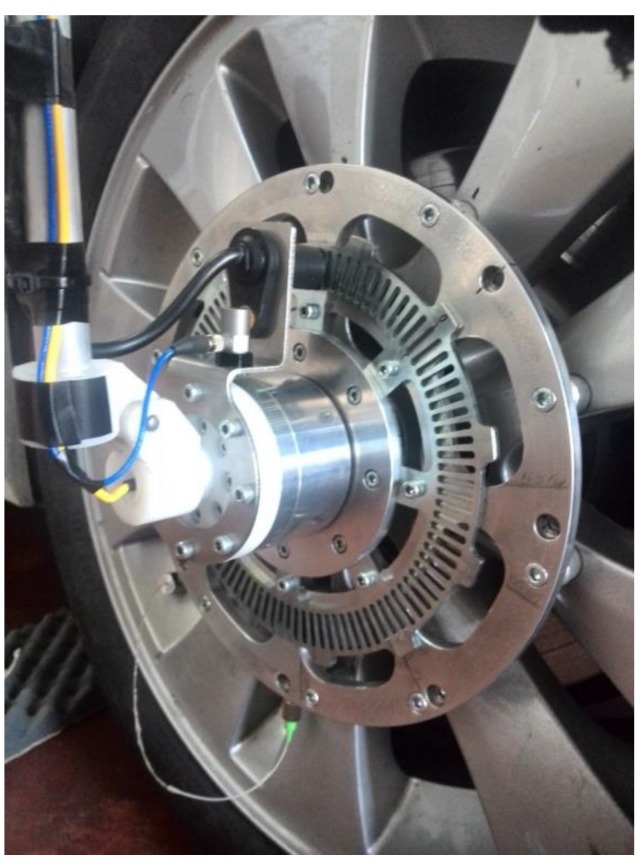
Wheel sensor device onboard with the piezo accelerometers and the wheel encoder.

This setup went beyond the current limits of the available technology, in terms of power supply and transmission data systems. The FBG sensors were supplied by a generator mounted externally to the tyre and integral to the car body. The generator produced the light source: the beam was sent to the optical fibre, embedded in the tyre carcass, through the rotary optical coupler. The optical signal sent on-board the tyre was modulated passing through the FBG sensor and was reflected back outside the tyre, thanks to the rotary optical coupler, which replaced any data transmission device. One of the main advantages of the solution was that the same physical light beam carried both the power to sensors and the information to the interrogator. Further advantages of the present setup arose from the material of these sensors, which had low rigidity and were capable of long-term acquisitions, even under the severe working conditions experienced by the tyres, and were very compatible with the rubber of the tyre. The size of the tyre adopted for the present was 215/45 R17, the tyre was installed on a prototype vehicle shown in Figure 4.

## 3. Rolling Resistance Model of a Rolling Tyre

### 3.1. Analytical Model for the Strain Distribution over the Tyre Surface

The design of a tyre, which results from the compromise between comfort and driving performances [7,20], demands theoretical models and experiments and it has to optimally combine rolling drag, tyre weight, directional stability, wet handling, ride comfort, steering feel and service life.

The theoretical investigation was the essential background to the following experimental campaign, it recalled the classical elasticity theory, focusing on the strain along the circumferences of the tyre, and pursued closed-form solutions. Accordingly, a model to characterize the strain was introduced as a model to estimate the rolling resistance of the tyre.

The model here discussed found its inspiration in Reference [8], which, more than providing analytical solutions to the problem, also locates the tyre–road contact patch, evaluates its length and recognizes where the progression between slipping and non-slipping takes place. Based on a similar approach, the present study further introduced and analysed the effect of the viscous damping on the deformation caused on the rolling tyre. The notations used in this paper are listed in the Table 1.

**Figure 5:**
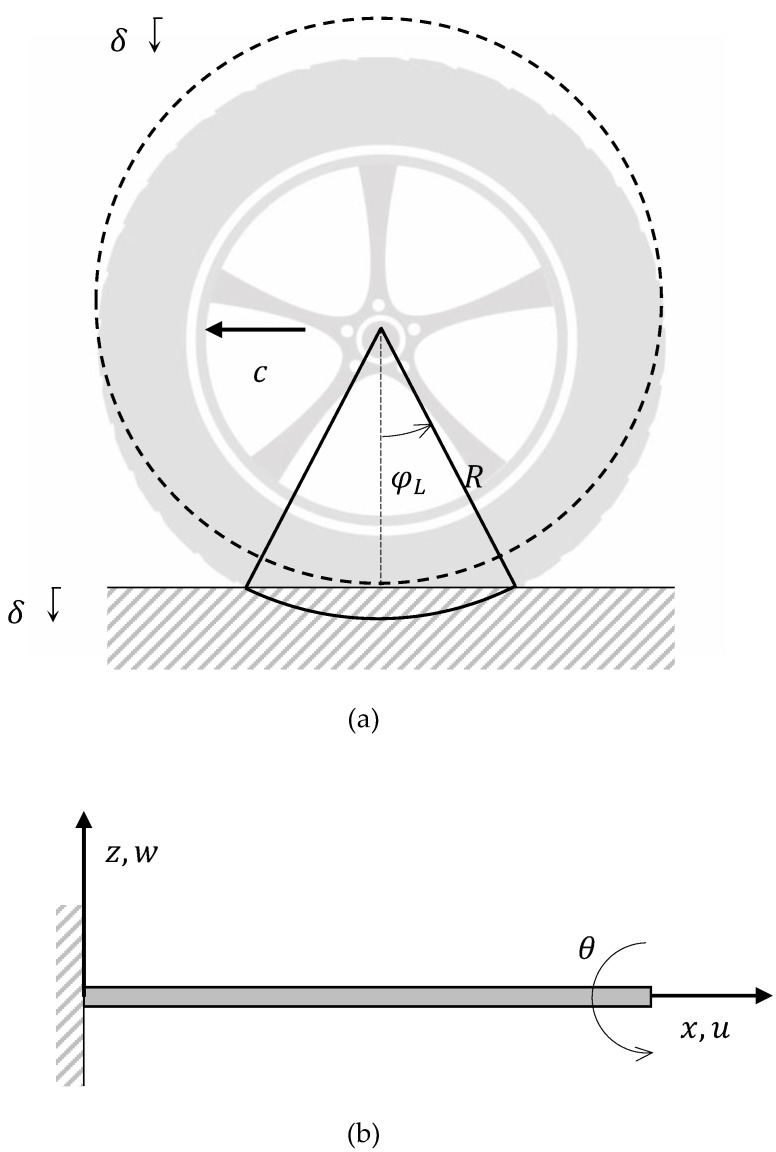
(**a**) Reference systems of the rolling tyre; (**b**) reference systems of the elastic beam.

The characterization of the analytical model requires the introduction of some hypothesis: i) the shape of the tyre, usually assumed to be a toroid (Figure 5a), is straighten out, ii) the tread is modelled as an infinite beam constrained to the elastic sidewall and iii) the sidewall itself is considered as a Winkler-type elastic base: the simplified model is shown in Figure 5b. The resulting differential equation of motion of the beam, expressed in terms of the transverse displacement *w* is:

(2) $$EJ\frac{\partial^{4}w\left(x,t\right)}{\partial x^{4}}+\mu \frac{\partial^{2}w\left(x,t\right)}{\partial t^{2}}+2\mu \omega_{d}\frac{\partial w\left(x,t\right)}{\partial t}+kw\left(x,t\right)=P\delta \left(x-ct\right)$$

with *x* the axial coordinate along the beam, as shown in the Figure 5b, and *t* is time variable, respectively, *EJ* represents the flexural stiffness, *µ* is the mass per unitary length, $\mu \omega_{d}$ represents dissipation effects, *k* is the Winkler foundation coefficient, *P* is the constant punctual vertical force, moving at constant speed *c* with respect to the points of the rolling tyre, and, eventually, δ(*x)* is the Dirac’s function. It is worth mentioning that *E* and μ in Equation (2) are considered constant while their values are also affected by the tyre temperature. However the characteristic time for significant temperature variations generally requires several minutes, $T_{temp}\sim 600s$, while the transverse displacement is characterised by a dominant period related to the tyre speed, which is much smaller than the former, i.e., $T_{rev}\sim 0.1s$ for a regular car tyre at 50 km/h. It is therefore allowed to neglect the temperature dependency of Equation (2), bearing in mind that the actual values of *E* and μ can be regularly updated once the tyre temperature is monitored: this is one of the main reasons why the multisensing setup adopted in the OPTYRE [35] also uses a dynamic temperature sensor. However, for the sake of simplicity in this first experimental campaign related to rolling resistance estimation and described in Section 4, the values of *E* and μ were not updated and their values listed in Table 1 refer to the start of the experiment with a measured tyre temperature of 20 °C.

It is also worth mentioning that the model here considered was linear elastic, i.e., constant Young’s modulus in Equation (2), in spite of the fact that tyre rubber is hyperelastic. This simplifying assumption was made to have a reasonable trade-off between two antithetic needs, such as the accuracy of predictions and the analytical/numerical efforts involved with the solution of the equations that govern the phenomenon. In fact, the stress–strain relationship remains linear also for the tyre rubber in the case of small and moderate deformations [36,37], diverging from the linear trend for larger strains. As a consequence, predictions made with the proposed model would be reliable in the case of moderate deformations of the tyre, i.e., in the case of constant speed or mild accelerating/braking torque, while the errors would grow for large deformations, which however are, generally, less frequent in daily driving conditions. 

The wave solution to the Equation (2) is obtained by the change of variable s=λ(x−ct), where $\lambda =\sqrt[{4}]{\frac{k}{4EJ}}$. The quasi-stationary solution of Equation (2) becomes the product between the static deflection $w_{0}=\frac{P}{8\lambda^{3}EJ}=\frac{P\lambda }{2k}$ and the nondimensional deflection $\tilde{w}\left(s\right)$. As a function of the single variable s, Equation (2) turns into a fourth-order ordinary differential equation:

(3) $$\frac{d^{4}\tilde{w}\left(s\right)}{ds^{4}}+4\alpha^{2}\frac{d^{2}\tilde{w}\left(s\right)}{ds^{2}}-8\alpha \beta \frac{d\tilde{w}\left(s\right)}{ds}+4\tilde{w}\left(s\right)=8\delta \left(s\right)$$

where:

(4) $$\alpha =\frac{c}{c_{cr}}=\frac{c}{2\lambda }\sqrt{\frac{EJ}{\mu }},\beta =\sqrt{\frac{\mu }{k}}\omega_{d}$$

and $c_{cr}$ is the beam critical speed:

(5) $$c_{cr}=2\lambda \sqrt{\frac{EJ}{\mu }}.$$

The solution to Equation (3) is achieved by introducing the integral Fourier transformations

(6) $$\begin{array}{c} \tilde{w}\left(s\right)=\frac{2}{a_{1}\left(D_{1}^{2}+D_{2}^{2}\right)}e^{-bs}(D_{1}^{}cos\;a_{1}s+D_{2}^{}sin\;a_{1}s),\mathrm{with}s\geq 0\\ \tilde{w}\left(s\right)=\frac{2}{a_{2}\left(D_{3}^{2}+D_{4}^{2}\right)}e^{bs}(D_{3}^{}cos\;a_{2}s-D_{4}^{}sin\;a_{2}s),\mathrm{with}s<0 \end{array}$$

and applying the boundary conditions:

(7) $$s\to \pm \infty :\;\tilde{w}\left(s\right)={\tilde{w}}^{\prime }\left(s\right)={\tilde{w}}^{\prime \prime }\left(s\right)={\tilde{w}}^{\prime \prime \prime }\left(s\right)=0$$

that leads to:

(8) $$\begin{array}{c} D_{1,3}=a_{1,2}b\\ D_{2,4}=b^{2}\mp \frac{1}{4}\left(a_{1}^{2}-a_{2}^{2}\right) \end{array}.$$

At last, the constants a and b are found under the hypothesis of light damping β≪1 as

(9) $$b=\sqrt{1-\alpha^{2}},a_{1,2}=\sqrt{1+\alpha^{2}\pm \frac{2\alpha \beta }{b}}.$$

It is apparent that, in case of a conservative tyre, for β=0, $a_{1}\equiv a_{2}=a=\sqrt{1+\alpha^{2}}$. The undamped expression of $\tilde{w}\left(s\right)$ according to Equations (7) is:

(10) $${\tilde{w}}_{ud}\left(s\right)=\frac{e^{-b\left|s\right|}}{ab}(a\;cos\;as+b\;sin\;a\left|s\right|)$$

which is an even function depending on the unique variable *s*.

The evaluation of the damped response is not straightforward to obtain and needs the expression as in Equation (6) to be expanded as a Taylor series around β up to the second order:

(11) $$\tilde{w}\left(s\right)\approx {\tilde{w}}_{ud}\left(s\right)-\mathrm{sign}\left(s\right)\frac{\alpha \beta }{ab}\left[\frac{1}{a}{\tilde{w}}_{ud}\left(s\right)+\frac{1}{b}\frac{e^{-b\left|s\right|}}{ab}\left(1+b\left|s\right|\right)(b\;cos\;as-a\;sin\;a\left|s\right|)\right].$$

Since the terms on the right-hand side in the square brackets are all even functions multiplied by the odd function sign(s), their product is itself an odd function:

(12) $$\psi \left(s\right)=-\mathrm{sign}\left(s\right)\left[\frac{1}{a}{\tilde{w}}_{ud}\left(s\right)+\frac{1+b\left|s\right|}{b}\frac{e^{-b\left|s\right|}}{ab}(b\;cos\;as-a\;sin\;a\left|s\right|)\right].$$

This implies that the damped deformation $\tilde{w}\left(s\right)$ is the result of the combination of two terms: the undamped response ${\tilde{w}}_{ud}\left(s\right)$ and a damped contribution, in which the effect of the damping appears only in the multiplying coefficient $\epsilon =\frac{\alpha \beta }{ab}$, linearly dependent on β. If it is reasonable to assume ϵ≪1, then the damped deformation emerges as a perturbation, in the shape of an odd function, that slightly alters the undamped response:

(13) $$\tilde{w}\left(s\right)\approx {\tilde{w}}_{ud}\left(s\right)+\epsilon \psi \left(s\right).$$

The stress–strain constitutive relationship provides the deformation of the beam:

(14) $$\varepsilon_{x}=-\frac{M}{EJ}y=-w^{\prime \prime }y$$

where $\varepsilon_{x}$ is the strain along the *x* direction and *y* the distance from the neutral axis. According to Equation (13), also the circumferential strain is expressed in terms of an even function, related to the undamped response, and a small perturbation, that assumes the form of an odd function:

(15) $$\varepsilon \left(s\right)\approx \varepsilon_{ud}\left(s\right)+\epsilon \varphi \left(s\right).$$

**Figure 6:**
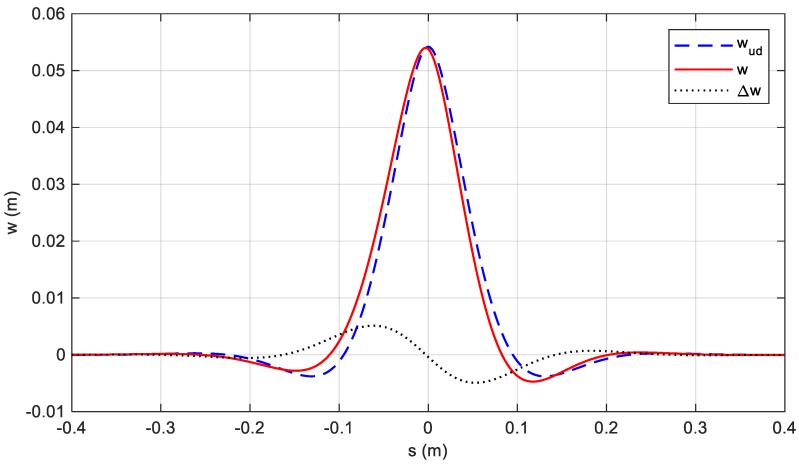
Undamped, damped vertical deflections and their difference in the function of the variables.

Figure 6 reports $\tilde{w}\left(s\right)$, ${\tilde{w}}_{ud}\left(s\right)$, calculated through Equations (6) and (10), respectively, and their difference $\Delta \tilde{w}\left(s\right)=\tilde{w}\left(s\right)-{\tilde{w}}_{ud}\left(s\right)$. The plot, based on the parameters presented in Table 2, confirms the accuracy of the model, given the resulting small amplitude odd function $\Delta \tilde{w}\left(s\right)$, consistent to Equation (13).

### 3.2. Analytical Model for the Rolling Resistance

It has already been mentioned how the contact patch undergoes a vertical load that is not perfectly centred, and it suffers an offset since the normal pressure is not uniformly distributed over the surface of the footprint; indeed, the trailing half is more unloaded with respect to the leading half. This phenomenon produces a torque, which operates in opposition to the forward motion, and it is mainly due to the hysteresis of the tyre, intrinsically associated with the deformation of the rubber during the rolling motion. This eventually generates the rolling resistance, and actually almost 90% of it is produced by the energy dissipated because of the deformation [2]. This implies that the main factors playing a role are the materials the tyres are composed of, the inflation pressure, the temperature, partially the speed of the car and in general the operating conditions.

With these premises, the present section was aimed at defining a measure of the energy loss because of the rolling resistance, based on few parameters that must be controllable and easily measurable, as the geometric features of the tyre and the strain along the circumferences of the tyre.

For this purpose, the work–energy relation between the amount of external work, the internal work and the kinetic energy of a deformable solid are presented. The amount of external work *P*(*V,t*) done on the deformed solid of volume *V*, confined by the surface *A* at the time *t* is:

(16) $$P\left(V,t\right)=\int_{V}\rho b\cdot vdV+\int_{A}T\cdot vdA$$

where *ρ* is the mass density in the considered configuration, **b** the body force per unit mass distributed over the volume *V*, **T** is the contact force per unit area or traction, ***v*** is the particle velocity at the point **x**. All the parameters in Equation (16) are functions of the time *t* and the configuration **x**, but the dependency has been concealed for the sake of notation. 

As long as it is reasonable to consider motions as infinitesimal, the traction **T** can be assumed to be the projection of the Cauchy stress tensor **σ** on the outgoing normal versor **n** to the surface A, namely, in Einstein notation, $T_{ij}=\sigma_{ij}n_{j}$. Applying the divergence theorem and expanding Equation (16), it becomes:

(17) $$P\left(V,t\right)=\int_{V}\left(\rho b_{i}v_{i}+\frac{\partial \sigma_{ij}v_{i}}{\partial x_{j}}\right)dV.$$

The linear momentum equilibrium equation, a function of the Cauchy stress, is:

(18) $$\rho \frac{\partial v_{i}}{\partial t}=\frac{\partial \sigma_{ij}}{\partial x_{j}}+\frac{\partial \sigma_{ij}}{\partial x_{j}}+\rho b_{i}.$$

Introducing Equation (18) into Equation (17) and employing the symmetry properties of the stress tensor, one obtains:

(19) $$P\left(V,t\right)=\int_{V}\left[\frac{1}{2}\rho \frac{\partial \left(v_{i}v_{i}\right)}{\partial t}+\frac{1}{2}\sigma_{ij}\left(\frac{\partial v_{i}}{\partial x_{j}}+\frac{\partial v_{j}}{\partial x_{i}}\right)\right]dV.$$

Eventually, the work-energy relation is achieved considering ${\dot{\varepsilon }}_{ij}=\frac{\partial \varepsilon_{ij}}{\partial t}=\frac{1}{2}\left(\frac{\partial v_{i}}{\partial x_{j}}+\frac{\partial v_{j}}{\partial x_{i}}\right)$ the strain rate tensor and combing Equation (19) and Equation (16):

(20) $$\int_{V}\rho b_{i}v_{i}dV+\int_{A}T_{i}v_{i}dA=\int_{V}\left(\frac{1}{2}\rho \frac{\partial \left(v_{i}v_{i}\right)}{\partial t}+\sigma_{ij}{\dot{\varepsilon }}_{ij}\right)dV$$

where *A* is the cross-section of the beam. Equation (20) states that the rate of external work on any part of the body equals the rate of increase of kinetic energy, e.g., the first right-hand side term, and the rate of internal work within that part, e.g., the second right-hand side term and which is often called the stress power. Generally, the stress power accounts for both stored and dissipated energy.

Since the dissipated power wants to be expressed as a function of the circumferential strain, the explicit definition of the strain tensor is required. As discussed in Section 2, the tread was modelled, based on the Newtonian fluid dissipation model, as an isotropic viscoelastic beam. It was further assumed that the tread was subjected to both longitudinal and flexural vibrations, according to Figure 5b, which implied vibrations along the x- and z-directions. Consequently, the strain tensor can be defined as:

(21) $$\begin{array}{c} \varepsilon_{11}=\frac{\partial u\left(x,t\right)}{\partial x}+z\frac{\partial \theta \left(x,t\right)}{\partial x}\\ \varepsilon_{13}=\varepsilon_{31}=\frac{\partial w\left(x,t\right)}{\partial x}+\theta \left(x,t\right) \end{array}.$$

In this set of equations, the indexes 1 and 3 correspond to *x* and *z* axis, respectively, and *w* represent the displacements along the longitudinal and vertical directions and *θ* is the rotation of the cross-section of the beam around the *y* axis, conform to the convention proposed in Figure 5a,b. It is apparent, the first of Equation (21) provides the total deformation of the beam along the *x* axis, due to the longitudinal stress (first term on the right-hand side) and due to the flexural contribution (second term still on the right-hand side). Finally, the second equation represents the shear deformation purely caused by the flexural displacement. When *E* and *G* are the Young’s and shear moduli and *η* the viscoelastic damping coefficient, the viscoelastic constitutive relationships appear to be:

(22) $$\begin{array}{c} \sigma_{11}=E\varepsilon_{11}+h{\dot{\varepsilon }}_{11}\\ \sigma_{13}=\sigma_{31}=G\varepsilon_{13}+h{\dot{\varepsilon }}_{13} \end{array}.$$

While the system embedded in the tyre, as presented in Section 2, was able to measure the longitudinal deformation, the experimental acquisition of the vertical displacement and of the cross-section rotation was not straightforward and its identification turned out to be rather demanding. For this reason, it was set aside in this work. This might induce an incomplete formulation; however, a reliable and meaningful one can still be provided under the following few assumptions:

- The beam is modelled according to the Euler–Bernoulli theory, for which $\theta \left(x,t\right)=-\frac{\partial w}{\partial x}$, the shear deformation and strain terms in Equations (21,22) are null and $\varepsilon_{11}=\frac{\partial u}{\partial x}-z\frac{\partial^{2}w}{\partial^{2}x}$.
- Transforming the coordinate system, i.e., the variable *x* is replaced with the curvilinear abscissa *Rφ*, and for a stationary motion, the new independent variable *s* becomes s=R(φ+ωt). The origin of the coordinate system has a motion integral with the tyre with angular speed *ω*. According to the convention in Figure 5a, the contribution of the angular speed has a positive sign, implying that when the rotation is positive, the velocity vector of the centre of the tyre has a direction opposite to the *x*-axis.

Thanks to these assumptions, it is now possible to write the longitudinal deformation in Equation (21) as a function of the inner tyre strain surface at $z=\frac{h}{2}$, i.e., $\varepsilon_{m}=\varepsilon_{11}\left(s,\frac{h}{2}\right)$. As the partial derivatives are transformed as $\frac{\partial }{\partial x}=\frac{d}{ds}$ and $\frac{\partial }{\partial t}=R\omega \frac{d}{ds}$, and ${\left(\right)}^{\prime }=\frac{d}{ds}\left(\right)$ for the sake of simplicity, it follows:

(23) $$\begin{array}{c} u^{\prime }=\varepsilon_{m}+\frac{h}{2}w^{\prime \prime };u^{\prime \prime }=\varepsilon_{m}^{\prime }+\frac{h}{2}w^{\prime \prime \prime }\\ \varepsilon_{11}=\varepsilon_{m}+\left(\frac{h}{2}-z\right)w^{\prime \prime };\frac{\partial \varepsilon_{11}}{\partial t}=R\omega \left(\varepsilon_{m}^{\prime }+\left(\frac{h}{2}-z\right)w^{\prime \prime \prime }\right) \end{array}$$

where the indication of the dependence on *s* is omitted.

The aim is to express the internal energy of the tyre as a function of the acquired strain and of the derivatives of the vertical displacement. This is obtained by substitution of Equation (23) into Equation (20), namely:

(24) $$K(\varepsilon_{m},\varepsilon_{m}^{\prime },w\prime ,w\prime \prime ,w\prime \prime \prime )=\int_{V}\frac{1}{2}\rho \frac{\partial (v_{i}v_{i})}{\partial t}dV=A{\left(R\omega \right)}^{3}\rho \int_{-\pi R}^{\pi R}(u\prime u\prime \prime +w\prime w\prime \prime )ds=\;=A{\left(R\omega \right)}^{3}\rho \int_{-\pi R}^{\pi R}(\varepsilon_{m}\varepsilon_{m}^{\prime }+\frac{h}{2}(\varepsilon_{m}w\prime \prime \prime +\varepsilon_{m}^{\prime }w\prime \prime )+\frac{h^{2}}{4}w\prime \prime w\prime \prime \prime +w\prime w\prime \prime )ds$$

With the same approach, the term related to the stress power in Equation (20) becomes:

(25) $$\begin{array}{c} S(\varepsilon_{m},\varepsilon_{m}^{\prime },w\prime \prime ,w\prime \prime \prime )=\int_{V}\sigma_{ij}{\dot{\varepsilon }}_{ij}dV=R\omega E\int_{V}\varepsilon_{11}\varepsilon_{11}^{\prime }dV+{\left(R\omega \right)}^{2}h\int_{V}\varepsilon_{11}^{\prime }{}^{2}dV=\\ R\omega EA\int_{-\pi R}^{\pi R}(\varepsilon_{m}\varepsilon_{m}^{\prime }+\frac{h}{2}(\varepsilon_{m}w\prime \prime \prime +\varepsilon_{m}^{\prime }w\prime \prime )+\frac{h^{2}}{3}w\prime \prime w\prime \prime \prime )ds\\ +{\left(R\omega \right)}^{2}hA\int_{-\pi R}^{\pi R}(\varepsilon_{m}^{\prime }{}^{2}+h\varepsilon_{m}^{\prime }w\prime \prime \prime +\frac{h^{2}}{3}w^{\prime \prime \prime 2})ds \end{array}$$

Applying the assumptions of the models of the previous section, i.e., in the case of moderate dissipation, both the vertical displacement *w* and the deformation of the circumference can be expressed as even functions affected by weak perturbations, in the shape of odd functions. To obtain the final expression of the dissipated power, one should evaluate each term Equation (24) and Equation (25) are composed of. Hence, starting from the first term on the right-hand side in Equation (24) it can be developed as follows:

(26) $$\int_{-\pi R}^{\pi R}\varepsilon_{m}\varepsilon_{m}^{\prime }ds\approx \int_{-\pi R}^{\pi R}\left(\varepsilon_{ud}^{}\varepsilon_{ud}^{\prime }+\epsilon \varepsilon_{ud}^{}\varphi^{\prime }+\epsilon \varepsilon_{ud}^{\prime }\varphi^{}+\epsilon^{2}\varphi \varphi^{\prime }\right)ds.$$

Since the integration domain is symmetric, the odd integrands result in a null contribution; instead, the even terms, being multiplied by the small parameter ϵ, generate modest contributions. Extending this scheme to all the other terms shows how the only remaining term on the right-hand side of Equation (20) is:

(27) $$K+S\approx {\left(R\omega \right)}^{2}\mathrm{hA}\int_{-\pi R}^{\pi R}\varepsilon_{m}^{\prime }{}^{2}\mathrm{ds}.$$

The result is the internal dissipated power becomes a function of the second power of the linear velocity of the tyre only. This implies that, given the circumferential strain, acquired through the embedded FBG sensors, the dissipated power can be easily evaluated through numerical integration, as discussed in the following section.

As a final comment, in the previous sections the importance of real-time identification methods has been underlined. According to this philosophy, the presented model becomes the answer to balance the need for a rapid algorithm, for real-time applications, and accurate mathematical characterization of the dissipated energy in a rolling tyre. 

Even though the achievement of these tasks is already challenging, there are a few more aspects that are worth further investigation. In particular we can mention the following points that deserve additional investigation: i) the contact mechanism between rough surfaces, based on the theory developed in Reference [14], ii) the presence of an interstitial fluid between the road and the tyre during the contact (tyre–water–road contact), which can be included thanks to the models presented in Reference [5], iii) the dynamics proper of the leading edge, according to the theory discussed in References [24,38], and iv) the sophisticated composite structure of the tyre, considered introducing high-order constitutive relationship, similarly to References [4,13]. 

## 4. Field Experiments and Discussion

**Figure 7:**
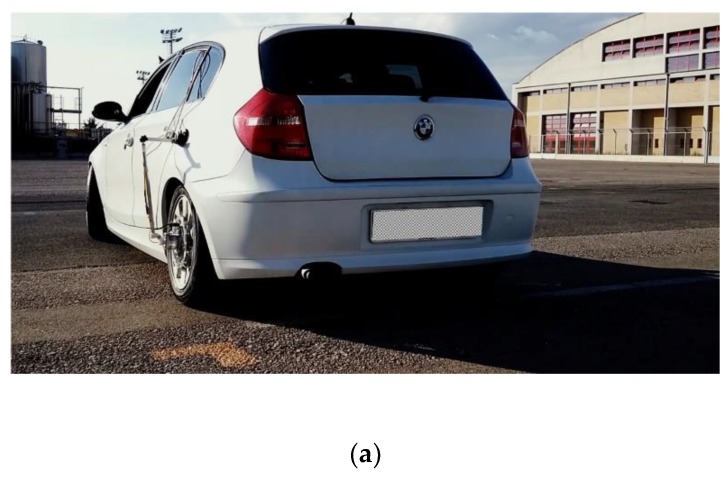

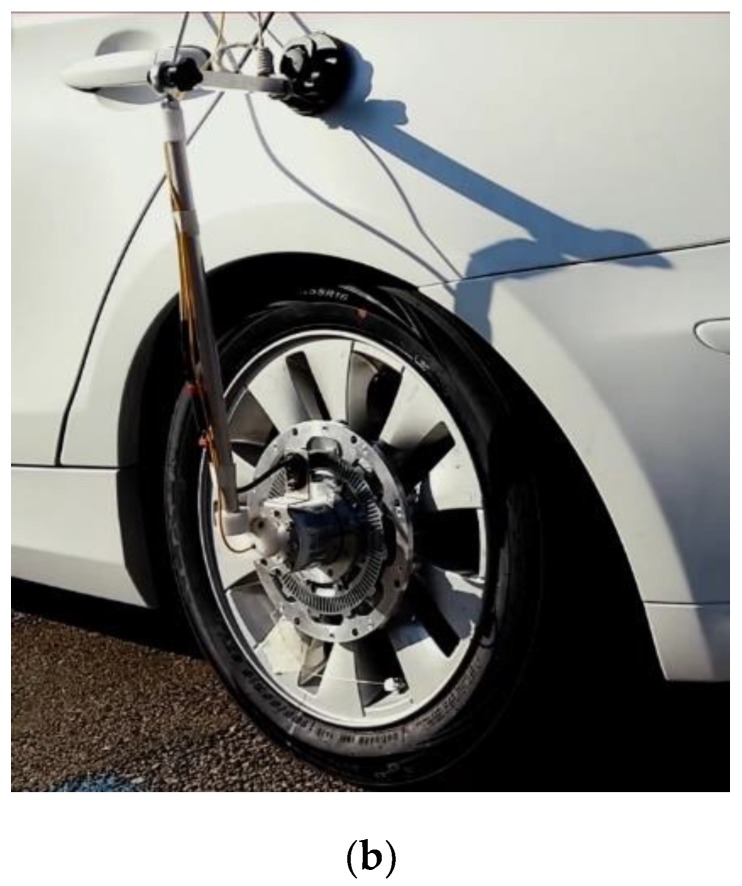
(**a**) The OPTYRE system mounted on the production car during the experimental campaign in operational condition; (**b**) a zoom of the measurement OPTYRE system in operational condition.

The experimental campaign is the focus of the present section. The experiments were carried out operating the car on urban and extra-urban roads. The acquisitions were made through two synchronized acquisition systems. The first was the optical interrogator connected to the FBG sensor while the second system captured data such as, the vertical acceleration of the wheel, the wheel encoder (see Figure 7a,b) and the data provided by the global position system (GPS). By combining the speed information detected by GPS and the encoder, it was possible to classify the data according to different speed regimes. During acquisitions, the vehicle retraced the same tracks several times, trying to keep the speed as constant as possible. Three passengers were sat in the car. The tyre was equipped with FBG sensors and the static weight on this tyre was measured and equal to 350 kg, and the tyre pressure was set at 2.2 atm.

**Figure 8:**
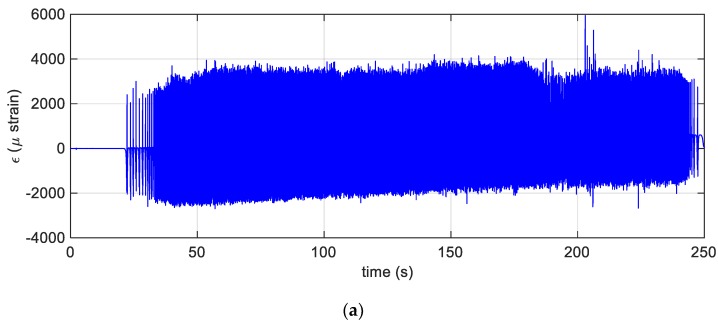

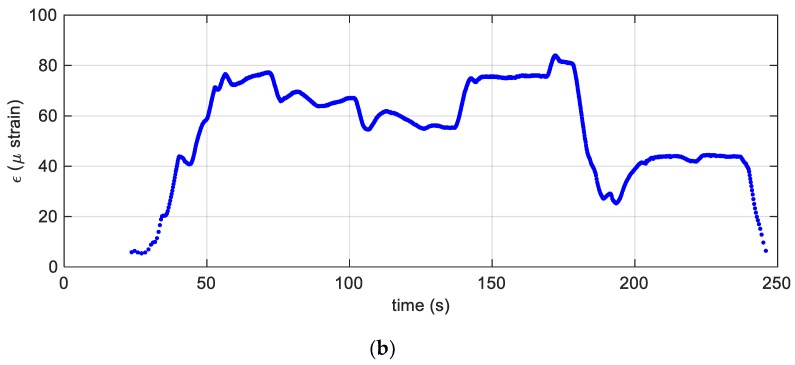
(**a**) The dynamic strain versus time; (**b)** the estimated tyre velocity plotted versus time.

The following figures (Figure 8a,b) report the achievements of the experiments performed under dynamic conditions. In particular, the time evolution of the inner surface circumferential strain is shown in Figure 8a. The signal was recorded during the phases of standing start, acceleration, uniform speed drive and braking. It is worth mentioning that the precision of the strain signal acquired allows also an accurate estimation of the tyre velocity: Figure 8b shows indeed an overlook of the trend of the tyre velocity. The speed was numerically evaluated applying 2^nd^ order centred differencing formula through a suitable Matlab^TM^ algorithm, namely the find peaks function, which uses the strain signal as input. The estimation is based on the fact that the maximum points of the strain are located about the centre of the footprint and that the relative distance between consecutive peaks is 2πR. This average, per revolution, tyre velocity turned out to be very well estimated, since the relative error with the average tyre velocity estimated by the encoder, described above, remained always below 1%. To this extent, it is important to recall that the encoder was primarily adopted for the real time identification of the tyre position [35], which helped the estimation of the tyre footprint and the residual grip as discussed in Reference [8], instead the tyre velocity can be easily estimated and with good accuracy by the FBG sensors only.

**Figure 9:**
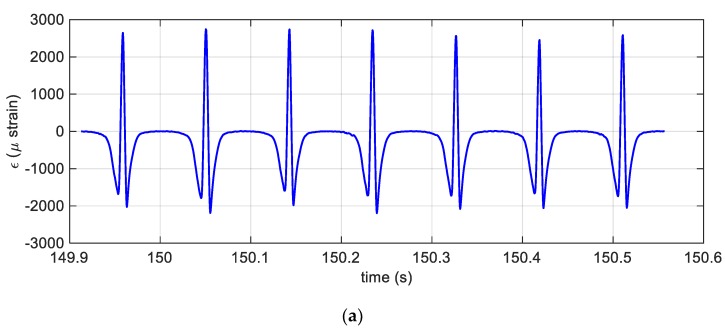

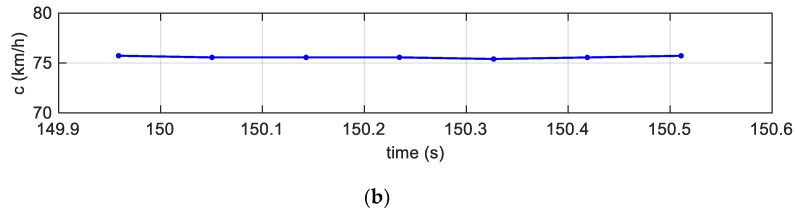
(**a**) Magnification of the strain plot in Figure 8a between 150 and 150.5 s with zero mean. (**b**) magnification of the strain plot in Figure 8 between 150 and 150.5 s.

Figure 9a,b display the circumferential strain during the uniform speed phase, the measured circumferential strain shown in Figure 9a appears to be in good agreement with the results of the analytical model shown in Figure 6, as explained also in more detail in Reference [8]; in addition, since the sensor was mounted on the inner surface of the tyre, the area of the contact patch was stretched.

In Figure 8a, it is possible to notice a weak drift of the strain average. This was due to the fact that at the beginning of the acquisition the tyre was cold, but the hysteresis loss, produced by the cyclical deformation of the tyre, generated a temperature increment. This aspect was further justified by the linear relationship between temperature and tyre inflation pressure: at the beginning of the cycle, FBG sensors appeared at the unloaded position, diametrically opposite to the centre of the contact patch; instead, at late times, the position changed because of the circumferential strain of approximately 600 μstrain. In first approximation, this temperature gradient could be set as about 10 °C for a thermal expansion coefficient of a generic rubber.

The model used in Equation (1), even though intrinsically related to the effects of the temperature, did not consider the mentioned temperature variation. Indeed, the model was applied to a time acquisition range in which the temperature reaches a stationary condition, identified through the stationary mean deformation trend ε. To enhance the used model, introducing a more accurate estimation of the tyre deformation due to thermal expansion at the inner line, it was advisable to equip the tyre with a further FBG sensor, detached from the inner line but close to the FBG sensor devoted to inner line deformation measuring. 

**Figure 10:**
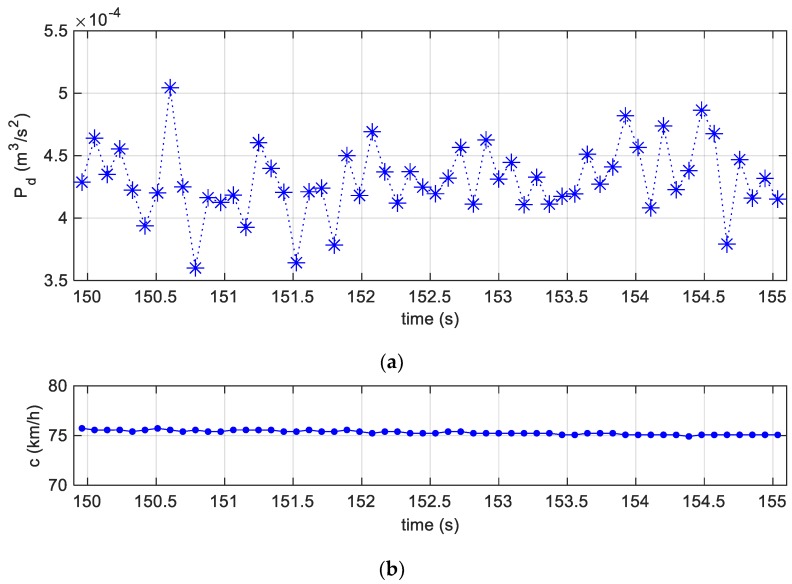
(**a**) Specific dissipated power between 150 and 155 s; (**b**) the uniform tyre speed over the selected time interval between 150 and 155 s.

The specific power $P_{d}=\left(K+S\right)/h$, based on Equation (27) can be defined as:

(28) $$P_{d}={\left(R\omega \right)}^{2}A\int_{-\pi R}^{\pi R}\varepsilon_{m}^{\prime }{}^{2}\mathrm{ds}$$

and it is displayed in Figure 10a, in which, given an almost uniform tyre speed over the selected time interval, see Figure 10b, the fluctuations are mainly due to irregularities of the road.

**Figure 11:**
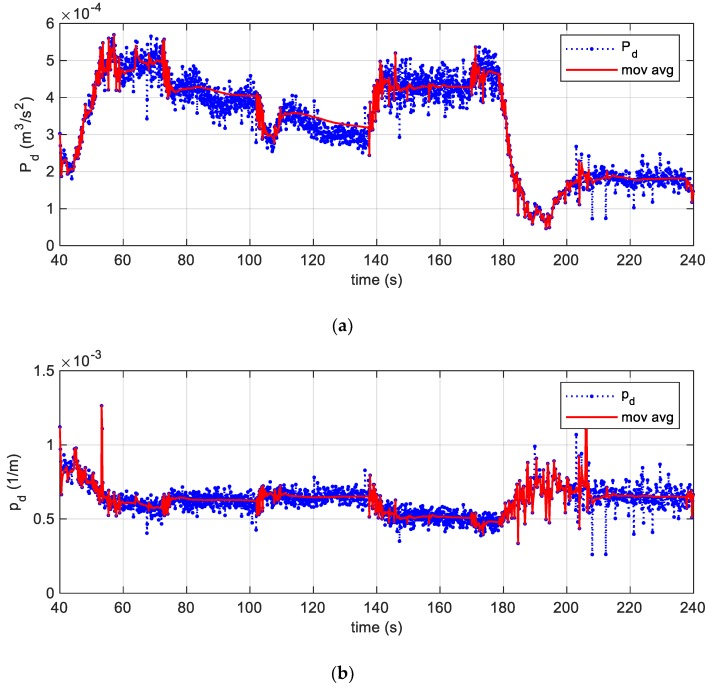

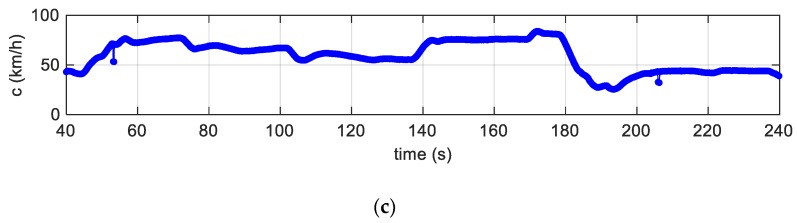
(**a**) The specific dissipated power and its moving average in blue and red lines, respectively; (**b**) the dissipation factor and its moving average; (**c**) the estimated velocity of the centre of the tyre.

The time evolution of $P_{d}$ over the entire acquisition interval is shown in Figure 11a. Data were cleaned by introducing the moving average, which was calculated in each interval where the tyre velocity could be assumed constant. To be considered constant, it was sufficient the requirement $c\left(t_{i}\right)-c\left(t_{i-1}\right)<0.08\;m/s$ was fulfilled. Note that the moving average filter was a simple low pass finite impulse response filter, which is commonly used for smoothing an array of sampled data/signal. It takes *N* samples of input at a time and takes the average of those *N*-samples and produces a single output point. It is a very simple low pass filter that comes in handy to filter unwanted noisy components from the original acquired data, while retaining a sharp step response. As the filter length *N* increases the smoothness of the output also increases, whereas the sharp transitions in the data are made increasingly less sharp. This filter has indeed excellent time domain response but a poor frequency response: this property makes it one of the best suited filters for time domain encoded signals, as the one here analysed in Figure 11a,b. However, the performances of the moving average are very poor for frequency domain encoded signals, with little ability to separate one band of frequencies from another.

The Figure 11b shows the trend of the dissipation factor $p_{d}$:

(29) $$p_{d}=\frac{P_{d}}{{\left(R\omega \right)}^{2}A}=\int_{-\pi R}^{\pi R}\varepsilon_{m}^{\prime }{}^{2}\mathrm{ds}$$

introduced so to delineate the relationship between the tyre speed (see Figure 11c) and the dissipated power.

**Figure 12:**
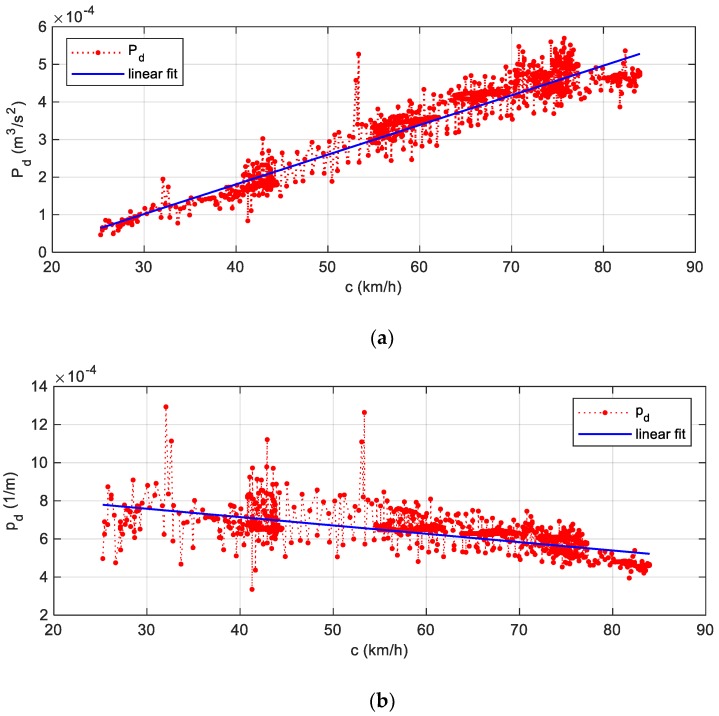
(**a**) The moving averages of the specific dissipated power $P_{d}$ evaluated in Figure 11a, sorted in ascending order in respect of the tyre velocity, along the x-axis; (**b**) the moving averages of the dissipation factor $p_{d}$ evaluated in Figure 11b, sorted in ascending order in respect of the tyre velocity, along the x-axis.

Eventually, the data, cleaned and smoothed, are shown in Figure 12a,b, arranged in ascending order with respect to the tyre velocity, i.e., the Figure 12a represents the specific power, while the Figure 12b the dissipation factor. As the dissipation factor linearly decreased with tyre speed, the specific power showed an opposite trend, regularly increasing along with a linear fit. Both linear fittings had a strong Pearson correlation coefficient, greater than 0.7. As a last consideration, the cloud of points around the regression line was due to road irregularities and inertia forces, related to transient actions, like hard braking and acceleration.

Finally, it is worth mentioning how the presented results can be related to the standard approach in the technical literature [39], which mainly deals with the rolling friction coefficient and losses in traction torque. Generally, the rolling resistance force can be defined using a rolling friction coefficient $\mu_{r}$, as follows:

(30) $$F_{r}=\mu_{r}F_{z}$$

where $F_{z}$ is the vertical load on the tyre, whose static load is known, and the dynamic load can be measured in real time by using a linear potentiometers sensor installed parallel on the suspension. The rolling resistance dissipated power is defined as follows:

(31) $$P_{d}=cF_{r}$$

where c is the tyre velocity. Once $P_{d}$ is estimated, as previously explained, and c is measured, the rolling friction coefficient can be easily evaluated:

(32) $$\mu_{r}=\frac{P_{d}}{cF_{r}}$$

The Equation (32) is the link between the usual approach to friction losses in tyre dynamics to the one proposed in this article.

## 5. Conclusions Remarks

Framed in the context of the tyre rolling phenomenon, this work presented an innovative technology for power dissipated identification through an ad hoc algorithm and experimental setup, conceived within the OPTYRE project.

The experimental apparatus was proven to be reliable and robust, being able to obtain long-lasting acquisitions, even in unfavourable conditions. The optical setup ensured high resolution and high accuracy strain signal measurements, without requiring a complicated power supply and data transmission systems. 

A semi-analytical method was here proposed for real-time sensing as it guaranteed the high speed required for real-time data investigation and the identification process: the proposed model struck indeed a good balance between a high computational velocity and an accurate analysis of the strain–grip relationship. The proposed model well characterized the interaction between tyre and road, starting from the definition of the dissipated power as a function of the tyre circumferential strain. It was also found how the dissipated power depended on the second power of the speed of the tyre. These results made the dissipated power easily evaluable, in each tyre revolution, given the measured circumferential strain acquired by the embedded FBG sensors. 

Both experimental and theoretical results confirmed that the OPTYRE technology allowed the real time identification of the tyre rolling resistance and, more generally, of the tyre–road grip conditions. Currently, a new multi-sensing setup [35], made of FBG sensors, a phonic wheel, a uniaxial accelerometer, and a dynamic temperature sensor, is employed and a wide experimental campaign is being conducted to get further insights in the intelligent tyre framework. 

## Figures and Tables

**Table 1:** Metamathematical notations.

Notations	Definition	Notations	Definition
x	Longitudinal space coordinate of the beam (m)	a,b	Constants of the bending beam solution
y	Transversal distance of the beam from the neutral axis (m)	ϵ=αβab	Perturbation coefficient associated with the nondimensional vertical displacement ψ of the damped beam
z	Vertical distance of the beam (m)	ε	Strain deformation
t	Time coordinate (s)	P	External power i.e., the work done on the deformed solid for unit time (Nm/s)
u(x,t)	Longitudinal displacement of the beam (m)	K	Kinetic power (Nm/s)
w(x,t)	Vertical displacement of the beam (m)	S	Stress power (Nm/s)
θ(x,t)	Rotation of the cross-section of the beam around *y* axis (rad)	V	Volume of the deformed solid (m^3^)
$w_{0}$	Static beam deflection (m)	A	Area of the deformed solid (m^2^)
E	Elastic modulus (Pa)	ρ	Density of the deformed solid (kg/m^3^)
G	Shear modulus (Pa)	b	Body force per unit mass distributed over the volume *V* (N/kg)
J	Second moment of area of the beam’s cross-section (m^4^)	v	Velocity vector of the particle (m/s)
μ	Mass per unitary length of the beam (kg/m)	T	Contact force per unit area or stress vector (N/m^2^)
$\omega_{d}$	Damping coefficient of the beam (1/s)	σ	Cauchy stress tensor (Pa)
k	Winkler elastic foundation (N/m^2^)	ε	Strain tensor
P	Vertical load (N/m)	n	Normal versor
c	Speed of load movement *P* or tyre speed (m/s)	η	Viscoelastic damping coefficient (Pa s)
$c_{cr}$	Critical speed of *c* (m/s)	φ	Angular tyre position (rad)
δ()	Dirac’s function	ω	Angular tyre speed (rad/s)
$\tilde{w}\left(\right)$	Nondimensional vertical displacement of the beam	R	Tyre radius (m)
${\tilde{w}}_{ud}$	Nondimensional vertical displacement of the undamped beam	h	Tyre thickness (m)
ψ	Nondimensional vertical displacement of the damped beam	$\varepsilon_{m}$	Longitudinal strain evaluated on the tyre contact surface
s	Nondimensional space coordinate	$p_{d}$	Dissipation factor (1/m)
α	Nondimensional stiffness of the beam	$P_{d}$	Power dissipated or specific power (m^3^/s^2^)
β	Nondimensional damping coefficient of the beam		

**Table 2:** List of the constant values (see the Reference [8] for more details).

Constant Values	
E = 5∙10^7^ N	Tyre Young modulus
J = 1.66∙10^−8^ m^4^	Beam area moment of inertia
µ = 1100 kg/m	Tyre mass per unit length
*k* = 7.790∙10^6^ N/m^2^	Elastic constant of the Winkler foundation
*R* = 0.3126 m	The unloaded radius of the tyre
*h_t_* = 0.018 m	Tyre section height
*L* = 0.05 m	Semi footprint length
M = 400∙9.81 N	Total load over the footprint
*C_x_* = 1.883∙10^6^ N/m	Longitudinal slip coefficient
*f_s_* = 0.8	Static tyre-road friction coefficient
*f_d_* = 0.6	Kinematic tyre-road friction coefficient
*c* = 11.11 m/s	Speed of the moving load
